# The Value of Auditing Surgical Records in a Tertiary Hospital Setting

**DOI:** 10.7759/cureus.21066

**Published:** 2022-01-10

**Authors:** Mahmoud Alqudah, Mohammed Aloqaily, Alexander Rabadi, Abdullah Nimer, Sufian Abdel Hafez, Amro Almomani, Nizar S Alkhlaifat, Ahmad Aldurgham, Ahmad Al-Momani, Zeyad Fraij, Wafi Aloqaily, Laith Bani Abedelrahman, Aya AlShati, Samir Jabaiti, Amjad Bani Hani, Mahmoud Abu Abeeleh

**Affiliations:** 1 Faculty of Medicine, The University of Jordan, Amman, JOR; 2 Faculty of Medicine, Jordan University Hospital, Amman, JOR; 3 Internal Medicine, Jordan University Hospital, Amman, JOR; 4 General Surgery, The University of Jordan, Amman, JOR; 5 General Surgery, Jordan University Hospital, Amman, JOR

**Keywords:** electronic medical records, surgical records, clinical audit, jordan university hospital, star score

## Abstract

Background: Assessing and improving quality of care should be of paramount importance to health care systems and providers. This study aimed to evaluate the quality of surgical records at the Jordan University Hospital.

Methods: We used the previously validated Surgical Tool for Auditing Records (STAR) to retrospectively evaluate the quality of surgical records of patients who underwent surgery in the general surgery department from 2016 to 2021. Total STAR and section-specific STAR scores were compared using the two independent sample Student’s ttest on SPSS Statistics, version 23 (IBM Corp, Armonk, NY).

Results: A total of 488 records were selected and evaluated using the STAR. The total STAR scores significantly improved steadily throughout the years compared to the baseline in 2016, reaching the highest in 2021. All domains had improved compared to the baseline except for anesthesia records that did not change from an already high baseline. The highest improvements between STAR domains were observed in Initial Clerking and Consent domains.

Conclusion: Our study demonstrates that significant improvements in the quality of surgical records can be achieved by simply using an electronic record entry system, personnel education, and systematic auditing.

## Introduction

Quality of care is a complex concept influenced by an elusive number of factors, some of which are easy to define and measure, while others are more abstract and socially constructed in nature [1]. Providing consistent, cost-effective, high-quality care to patients is a core objective all medical institutions strive to accomplish. Therefore, all efforts directed towards assessing and improving quality of care should be of paramount importance to health care systems and providers. While the perception of quality is not necessarily an accurate representation of actual quality provided, it is justified to pursue an improvement in quality parameters we can empirically change. One way to measure the quality of care provided to patients is by assessing the documentation quality of their medical information [2,3].

High-quality documentation should be comprehensive yet efficient focusing on presenting valuable information without becoming a barrier to proper care by wasting time and resources [4]. Medical records are an integral part of the health care system; they form the basic grounds for patients’ management, provide substantial data for medical research and protect medical professionals from legal liability [5-7]. While the traditional paper-based medical records are still widely used, electronic medical records (EMRs) have been increasingly incorporated in many facilities around the world, and shown to improve the quality of care compared to their paper-based counterpart [8,9].

A clinical audit is a quality improvement process in which clinical practice is evaluated against certain standards representing the ideal practice sought after to identify areas of shortcomings and implement positive change [10]. Several tools have been developed for auditing clinical records; the CRABEL (developed by CRAwford-BEresford-Lafferty) scoring system is one such tool that can be applied to any in-patient specialty [11]. The Surgical Tool for Auditing Records (STAR) is a modified version of the CRABEL system that is more tailored to surgical records’ auditing, and has been used in other studies with promising improvements [12-14].

Jordan University Hospital (JUH), Amman, Jordan, is a tertiary care teaching hospital that serves a large population of patients in Amman and Jordan. Despite it being a leading research center in the region, it is unfortunate that few clinical audits have been published in the literature [13,15]. This lack of clinical audits as a quality improvement initiative extends to other institutions in Jordan and the Middle East region with few examples to spare [16-22]. Hence, more awareness of the clinical value and research potential of auditing current practices should be raised and acted upon. Our study aims to evaluate and improve the quality of surgical records’ documentation in our hospital by performing an audit cycle using the STAR scoring system.

## Materials and methods

The study involved surgical records for patients undergoing various surgeries at JUH. JUH is the vast pioneer academic hospital in Jordan. Furthermore, it serves more than four million patients by being located in the center of Jordan [23]. JUH houses 600 beds and treats more than half a million patients annually while conducting about 20,000 surgical procedures every single year [24]. Recently, JUH installed its EMR system as an adjunct to the hospital’s paper-based system from 2009 to present.

STAR was chosen to conduct a retrospective evaluation and audit the quality of surgical records between 2016 and 2021 [14].The STAR system is a significantly reliable (Cronbach's α: 0.959) alternative to CRABEL for medical record keeping. The tool is composed of 50 components allocated into six domains of different weight allotments including Initial Clerking (10 items; 20%), Subsequent Entries (8 items; 16%), Consent (7 items; 14%), Anesthetic Record (7 items; 14%), Operative Record (9 items; 18%), and Discharge Summary (9 items; 18%). The tool initially requires a minimum of 20 records to be utilized. The total score for each evaluated note is calculated based on the following formula: (50 - deducted points) x 2. The Subsequent Entries domain is calculated by averaging out the final score over the number of up to four entries post the initial entry. Likewise, the total STAR score is the average of all evaluated notes [14].

Records of patients who underwent surgery between 2016 and 2021 were chosen at random given that they were admitted for at least one day, had no delay in surgery, and their surgery was not categorized as day surgeries. A pilot assessment was first applied on 20 records to train two authors on how to manage surgical records using the STAR and troubleshoot any queries that may surface between authors in STAR’s concept interpretation. The two authors individually evaluated 488 surgical records located within the hospital’s EMR and then were cross-matched. Any conflict between the two investigators was resolved by a third senior author delegating through unbiased lens.

Traditionally, all medical faculty and staff among all the 64 specialties in JUH receive annual workshops to improve documentation and write concise notes. The purpose of the audit was to assess the quality transition of surgical notes by comparing their total STAR scores and percentages of deficiency within specific areas of note-taking. The collected data were reported as frequencies [n (%)], and means ± standard deviations wherever applicable. Pre- and post-audit total STAR and section-specific STAR scores were compared using the two independent sample Student’s t test. A p-value of less 0.05 at a confidence interval of 95% was considered statistically significant. All data cleaning and statistical analyses were conducted on SPSS Statistics, version 23 (IBM Corp, Armonk, NY).

Review and acceptance were done by the JUH’s Institutional Review Board (IRB) and the University of Jordan’s research ethics committee. The processes within the study’s protocol adhere to the guidelines of the Declaration of Helsinki (1996).

## Results

**Figure 1:**
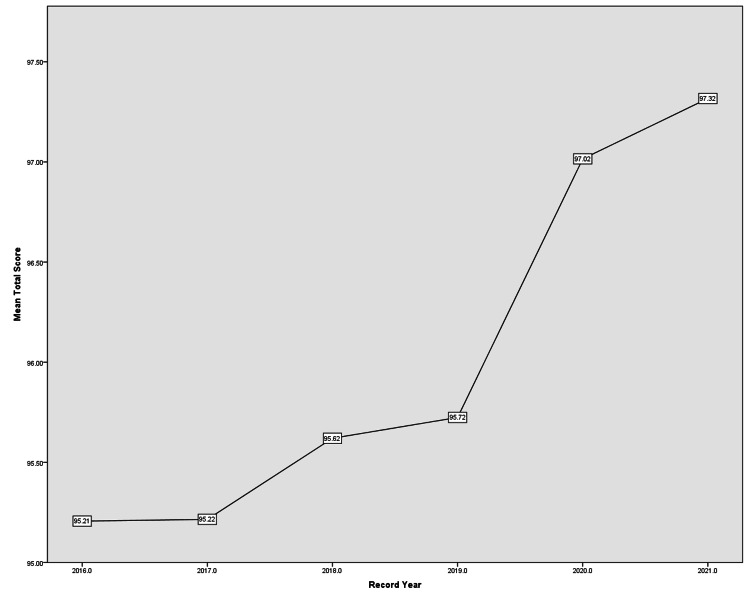
Mean STAR score STAR, Surgical Tool for Auditing Records

The study included 488 surgical records of patients undergoing surgery throughout the following years: 2016 (71), 2017 (82), 2018 (86), 2019 (81), 2020 (88), and 2021 (80). The mean total STAR score for the years, the overall STAR score and section-specific STAR scores for all the included years are demonstrated in Table 1. Using 2016 as a baseline, total STAR scores of records of the later years kept improving significantly at a continuous pace along the years where the greatest increase was between years 2019 and 2020 with an unprecedented all-time high total STAR score in 2021 compared to previous years (Figure 1). Each of the domains and their respective subdomains had the following results.

**Table 1:** STAR scores among surgical records from 2016 to 2021 STAR, Surgical Tool for Auditing Records ^*^Identifies a significant mean difference when compared to the 2016 baseline at p-value < 0.05. ^!^Identifies a significant mean difference when compared to the 2018 baseline at p-value < 0.05. ^#^Identifies a significant mean difference when compared to the 2019 baseline at p-value < 0.05. ^$^Identifies a significant mean difference when compared to the 2020 baseline at p-value < 0.05.

	2016	2017	2018	2019	2020	2021
Initial Clerking score	96.1±2.4	96.5±2.1	97.2±1.6*	96.7±2.1	98.5±1.1*!#	99.3±1.2^*,!,#,$^
Subsequent Entries score	95.8±3.9	95.0±2.3*	95.4±1.8*	95.4±1.9*	98.7±1.4*!#	99.1±1.2^*,!,#^
Consent score	94.3±1.5	94.6±1.3	95.5±1.3*	95.8±1.5*	96.5±1.1*!#	96.8±1.2^*,!,#^
Anesthetic Record score	98.0±0.0	98.0±0.0	98.0±0.0	98.0±0.0	98.0±0.0	98.0±0.0
Operative Record score	91.9±2.5	91.6±2.2	91.8±1.9	92.0±2.2	93.0±2.4*!#	93.2±2.2^*,!,#^
Discharge Summary score	95.2±1.8	95.5±1.4	95.7±1.4	96.4±1.4*!	97.5±1.6*!#	97.5±1.8^*,!,#^
Total score	95.2±1.2	95.2±0.8	95.6±0.7	95.6±0.7*	95.7±0.8*!#	97.3±0.9^*,!,#,$^

Initial Clerking score

**Figure 2:**
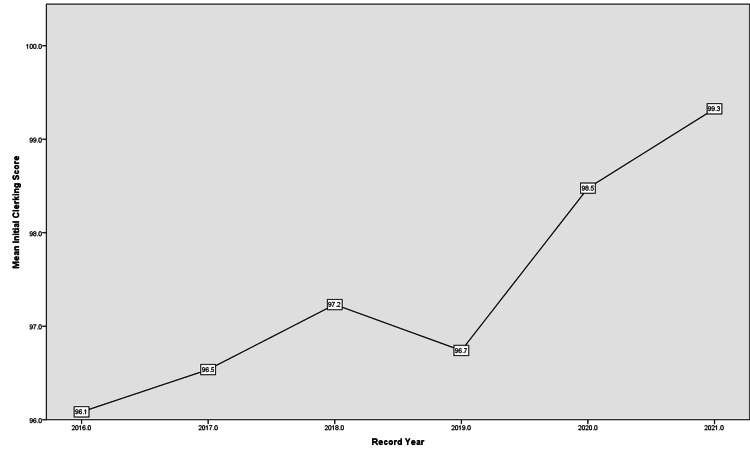
Initial Clerking score

The mean initial clerking score showed continuous improvement from 96.1 in 2016 to 99.3 in 2021 with only a slight decrease in 2019 (Figure 2). Hospital Number, Referral Source, Consultant, Date/Time, and Name/Bleep/Post subdomains reached 100% by 2020 and remained so by 2021. The rest of the subdomains had their highest score in 2021 indicating overall improvement over time in this domain.

Subsequent Entries score

**Figure 3:**
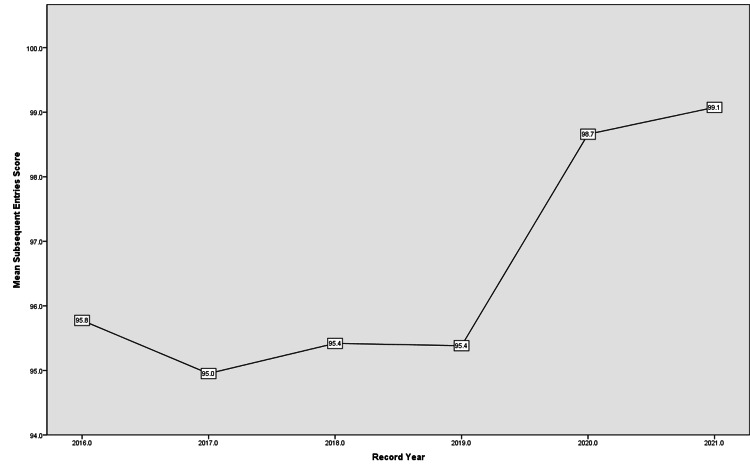
Subsequent Entries score

The mean Subsequent Entries score remained fluctuating around 95.4 from 2016 till 2019 but rose in the following years (Figure 3).

Consent score

**Figure 4:**
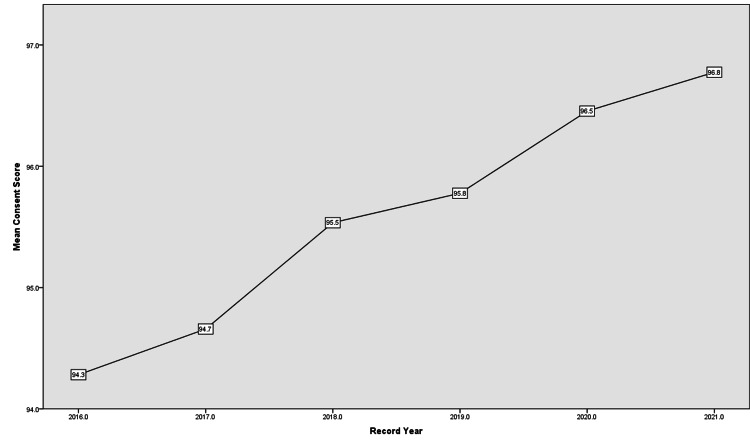
Consent score

For the mean Consent score, it had consistency in increasing over the years (Figure 4). Name/Number/Date, Operation, and Benefits reached 100% by 2020 and remained so by 2021 while Signature lost the perfect three-year streak of 100% in 2021 to become 98.8%. Risk/Complications had the greatest improvement over the five-year period from 8.5% to 93.8% unlike Side and Site in full words that remained the lowest subdomain.

Anesthetic Record score

**Figure 5:**
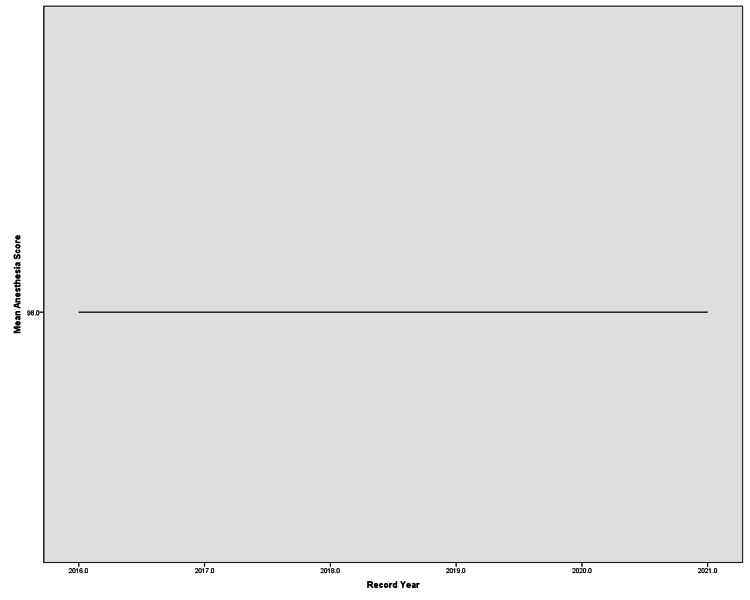
Anesthetic Record score

Meanwhile, the mean Anesthetic Record score was fixed at 98 in all the years examined (Figure 5). Overall, 2020 and 2021 had the highest scores in this domain. By 2021, Monitoring Data and Name of Anesthetist/Consultant reached 100% while Post-op Instructions had a fixed 100% in all years.

Operative Record score

**Figure 6:**
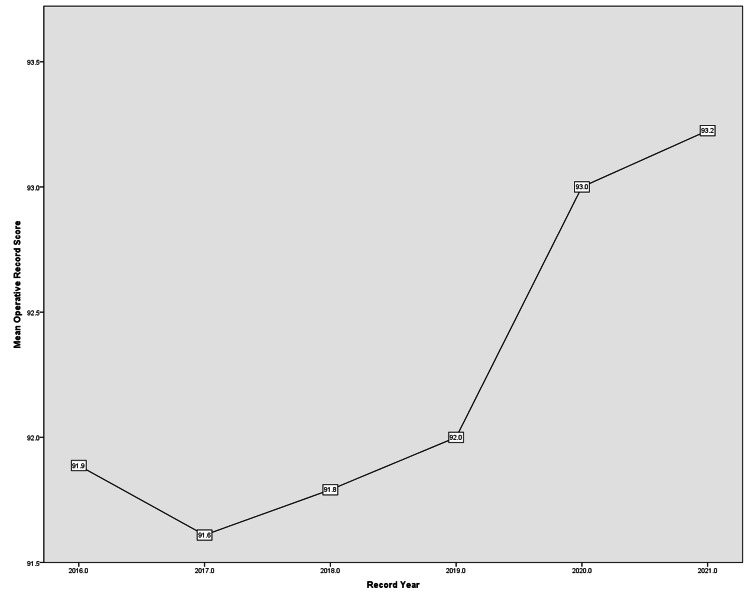
Operative Record score

In regard to the mean Operative Record score, only a slight increase was observed between 2016 and 2021 (Figure 6). Name/Number/Date had a 100% streak in all years with Operating Surgeon maintaining the same trend till 2021 when it decreased from 100% to 98.8%. However, Prosthetics/Serial Number plummeted to 2.5% in 2021 after a three-year rank of 100%, unlike Post-op Instructions that had the lowest scores of all domains.

Discharge Summary score

**Figure 7:**
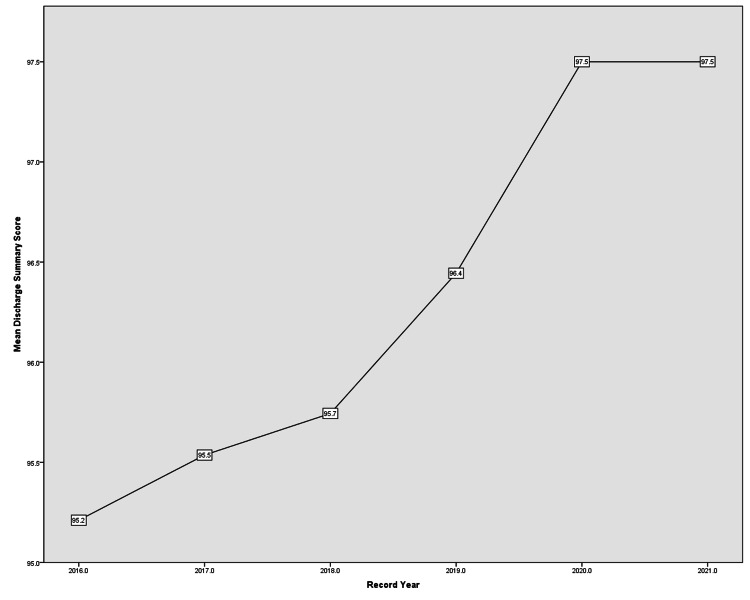
Discharge Summary score

Finally, the mean Discharge Summary score had a frequent incline but reached a plateau in 2020 and 2021 (Figure 7). Admission/Discharge dates and Discharge Consultant had a fixed 100% in all years preceding Diagnosis and Operation/Procedure that only reached 100% in 2020 and 2021. As for Complications and Medications on Discharge, relatively lower scores were observed yet promising increments happened along the years examined. All subdomains showcased previously are illustrated in Table 2.

**Table 2:** Detailed STAR scores STAR, Surgical Tool for Auditing Records

Domains	Subdomains	2016	2017	2018	2019	2020	2021
Initial Clerking	Name	71 (100%)	82 (100%)	86 (100%)	81 (100%)	88 (100%)	80 (100%)
	Hospital number	71 (100%)	77 (93.1%)	86 (100%)	80 (98.8%)	88 (100%)	80 (100%)
	Referral source	59 (83.1%)	83.1 (98.8%)	84 (97.7%)	75 (92.6%)	88 (100%)	80 (100%)
	Consultant	70 (98.6%)	80 (97.6%)	86 (100%)	81 (100%)	88 (100%)	80 (100%)
	Date/time	47 (66.2%)	61 (74.4%)	66 (76.7%)	62 (76.5%)	80 (90.9%)	79 (98.8%)
	Working diagnosis	59 (83.1%)	67 (81.7%)	77 (89.7%)	77 (89.5%)	69 (85.2%)	79 (98.8%)
	Investigations/results	10 (14.1%)	7 (8.5%)	9 (10.5%)	8 (9.9%)	32 (36.4%)	55 (68.8%)
	Management plan	51 (71.8%)	68 (82.9%)	80 (93.0%)	67 (82.7%)	88 (100%)	79 (98.8%)
	Allergies recorded	62 (87.3%)	77 (93.9%)	85 (98.8%)	77 (95.1%)	85(96.6%)	79 (98.8%)
	Name/bleep/post	70 (98.6%)	81 (98.8%)	82 (95.3%)	76 (93.8%)	88 (100%)	80 (100%)
Consent Form	Name/number/date	70 (98.6%)	82 (100%)	86 (100%)	80 (98.8%)	88 (100%)	80 (100%)
	Operation	69 (97.2%)	81 (98.8%)	86 (100%)	80 (98.8%)	88 (100%)	80 (100%)
	Side and site in full words	18(25.4%)	25 (30.5%)	21 (24.4%)	18 (22.2%)	25 (28.4%)	23 (28.7%)
	Risk/complications	6 (8.5%)	11 (13.4%)	51 (59.3%)	67 (82.7%)	86 (97.7%)	75 (93.8%)
	Signature	70 (98.6%)	80 (97.6%)	86 (100%)	81 (100%)	88 (100%)	79 (98.8%)
	Name/bleep/post	60 (84.5%)	76 (92.7%)	80 (93.0%)	70 (86.4%)	84 (95.5%)	79 (98.8%)
	Benefits	71 (100%)	82 (100%)	86 (100%)	81 (100%)	88 (100%)	80 (100%)
Anesthetic Record	Name of anesthetist/consultant	67 (94.4%)	82 (100%)	82 (95.3%)	75 (92.6%)	88 (100%)	80 (100%)
	Pre-op assessment	67 (94.4%)	81 (98.8%)	84 (97.7%)	81 (100%)	78 (88.6%)	72 (90.0%)
	Drugs and doses	60 (84.5%)	73 (89.0%)	68 (79.1%)	71 (87.7%)	81 (92.0%)	76 (95.0%)
	Monitoring data	68 (95.8%)	82 (100%)	85 (98.8%)	79 (97.5%)	87 (98.9%)	80 (100%)
	IVI given	55 (77.5%)	74 (90.2%)	76 (88.4%)	73 (90.1%)	83 (94.3%)	75 (93.8%)
	Name/signature	68 (95.8%)	80 (97.6%)	85 (98.8%)	76 (93.8%)	88 (100%)	78 (97.5%)
	Post-op instructions	71 (100%)	82 (100%)	86 (100%)	81 (100%)	88 (100%)	80 (100%)
Operative Record	Name/number/date	71 (100%)	82 (100%)	86 (100%)	81 (100%)	88 (100%)	80 (100%)
	Operating surgeon	71 (100%)	82 (100%)	86 (100%)	81 (100%)	88 (100%)	79 (98.8%)
	Diagnosis post-op	24 (33.8%)	19 (23.2%)	19 (22.1%)	14 (17.3%)	24 (27.3%)	18 (22.5%)
	Description of findings	53 (74.6%)	63 (76.8%)	73 (84.9%)	68 (84.0%)	77 (87.5%)	70 (87.5%)
	Details of tissues removed	46 (64.8%)	48 (58.5%)	52 (60.5%)	55 (67.9%)	63 (71.6%)	58 (72.5%)
	Details of sutures used	14 (19.7%)	13 (15.9%)	11 (12.8%)	18 (22.2%)	51 (58%)	58 (72.5%)
	Prosthetics/serial number	71 (100%)	2 (2.4%)	86 (100%)	81 (100%)	88 (100%)	2 (2.5%)
	Post-op instructions	6 (8.5%)	2 (2.4%)	9 (10.5%)	9 (11.1%)	6 (6.8%)	2 (2.5%)
	Surgeon/signature	66 (93.0%)	82 (100%)	84 (97.7%)	78 (96.3%)	88 (100%)	80 (100%)
Discharge Summary	Name/number/address	67 (94.4%)	81 (98.8%)	86 (100%)	81 (100%)	88 (100%)	80 (100%)
	Admission/discharge dates	71 (100%)	82 (100%)	86 (100%)	81 (100%)	88 (100%)	80 (100%)
	Discharge consultant	71 (100%)	82 (100%)	86 (100%)	81 (100%)	88 (100%)	80 (100%)
	Diagnosis	65 (91.5%)	81 (98.8%)	84 (97.7%)	81 (100%)	88 (100%)	80 (100%)
	Pertinent investigations/results	62 (87.3%)	62 (75.6%)	70 (81.4%)	66 (81.5%)	82 (93.2%)	62 (77.5%)
	Operation/procedure	69 (97.2%)	80 (97.6%)	84 (97.7%)	80 (98.8%)	88 (100%)	80 (100%)
	Complications	2 (2.8%)	3 (3.7%)	2 (2.3%)	17 (21.0%)	26 (29.5%)	24 (30.0%)
	Medications on discharge	12 (16.9%)	10 (12.2%)	12 (14.0%)	20 (24.7%)	48 (54.5%)	54 (67.5%)
	Follow-up	50 (70.4%)	72 (87.8%)	81 (94.2%)	78 (96.3%)	86 (97.7%)	79 (98.8%)

## Discussion

The STAR scoring system is an auditing tool meant to measure the quality of record-keeping in health institutions. In this audit, the mean total STAR score was measured for the last six years and showed consecutive improvement in every year with year 2021 having the highest total score of 97.3±0.9 with statistical significance compared to almost all previous years individually as shown in Table 1. This improvement complements the previous literature since the first development of STAR score by Tuffaha et al. who reported an increase in the total STAR score from 83.4 to 97.6 after the audit cycle was complete [14]. Basu et al. also reported an improvement in the STAR score from 87 in the first cycle to 93 in the second one [25]. It is worth noting that the relative improvement in JUH was less than both these audits’ that might be due to the high baseline STAR score in JUH that started at 95.2 in 2016.

Looking at the individual sections of the tool, it is clear that improvement is present in every domain except for anesthesia recordings that show a clear plateau at a score of 98; this might be because that even before the start of this audit, anesthesia records were standardized into a form required to undergo any elective surgery, and is consistent with the finding of Mafrachi et al. who noticed the same plateau while studying anesthesia STAR scores in JUH [13]. This suggests that standardizing a form for record entry is useful especially with EMRs, which is consistent with previously reported results [26,27].

The consent score showed a significant improvement after adding required items to the consent form to undergo elective surgeries in 2018 including expected risks and complications of any operation. Risk documentation in consent forms went from 8.5% in 2016 to 93.8% in 2021 as shown in Table 2, presenting the biggest improvement found in any item, and illustrating the level of change a systemic policy can accomplish if applied well. This is also seen in the discharge-summary-reported complications that went from 2.8% in 2016 to 30% in 2021; the low percentage of reporting in the discharge summary might be because residents are more likely to report it in progress notes. The improvement discussed above is an important measure that reflects the efficacy of a cyclic audit approach that includes increasing residents’ awareness about record entry and keeping [28].

An effective step taken to improve the quality of record entry as well as retrieval is the application of an electronic record system with a separate field for each element that is required to be filled in order to proceed. This forcing function has been previously shown to be an effective approach in the literature [29].

Proper medical documentation contributes to the integrity and quality of health care systems in ways that extend beyond its direct role in patient care. It provides a legally credible account of the events and interactions between patients and providers; this includes not only the assessment and medical interventions accepted by patients and their informed consent, but also those they rejected [30]. This proves to be of great significance in claims of malpractice, negligence, and health care fraud and abuse since the judiciary system relies heavily on collecting any available documented information [7,30-33]. Hence, proper documentation is essential in protecting medical providers and their programs. The pressing nature of this aspect of healthcare is illustrated by data such as those from the 2016 American Medical Association benchmark survey that showed that 34% of all physicians and over 63% of general surgeons have been sued at least once [34]. A potential downside that fears of lawsuits could present is the exhaustive and inefficient documentation approach some physicians might follow to avoid legal liability that comes at the expense of patient care [4].

Old records were scanned and implemented within the new archives. The literature has shown that EMRs had fewer missing items and saved at least 20% on the time of retrieval [35]. However, some studies suggested that due to the lack of a clear gold standard and comparative objective measures, it is recommended (when possible) to rely on both EMRs and paper-based records [36].

Since the development of the CRABEL score that aimed to provide a quantitative base measure to compare the quality of medical records, new tools have been emerging including the STAR score and even newer scores such as the Surgical Hospital Audit of Record Keeping (SHARK) score [11,37]. Both of these tools were designed according to the Royal College of Surgeons guidelines to overcome potential problems in the CRABEL score, such as the unequal weight for each set of items that could lead to lack of specificity, as well as its inability to maintain the initial improvement in subsequent cycles [37].

Even though STAR showed a good reliability and validity, it does portray all the variables important to surgical patients. The use of this tool is only limited to surgical specialties as surgeons tend to be more thorough to avoid legal issues. It is worth pointing out that misplacing the data within the patients’ progress notes can confuse as important items might be noted but not in their designated fields. The STAR score shows a particular design that has its own limitation due to its inability to detect major areas of deficiencies hidden within the vague nature of its concepts, without focusing on details. However, our strengths lie in our rigorous methodology, as we had surveyed a random sample of medical records that is average in number, using a well-validated tool within the literature with excellent reliability and low inter-observer variation.

## Conclusions

In the light of the above, this audit illustrates that the combined use of a specified electronic record entry system along with educating medical personnel on its use and the importance of record-keeping showed a significant improvement in its quality, which was evident by the use of the STAR score. This could lead to a firmer ground to rely on regarding medico-legal cases leading to the possible cost reduction. However, it is still not clear whether the use of such tools in audit cycling will lead to a decrease in the medico-legal burden. Additional audit cycles and quality improvement efforts regarding surgical records are still needed to reach higher standards of patient safety and quality of care.
